# Using the new electronic frailty index (eFI2) to aid frailty identification and management in primary care

**DOI:** 10.3399/bjgp25X742473

**Published:** 2024-05-30

**Authors:** Danielle Nimmons, Andrew Clegg, Kate Walters

**Affiliations:** Research Department of Primary Care and Population Health, Centre for Ageing and Population Studies, UCL, London; Clinical Director, Spen Health and Wellbeing Primary Care Network.; Academic Unit for Ageing & Stroke Research, University of Leeds, Bradford Teaching Hospitals NHS Foundation Trust, Bradford.; Research Department of Primary Care and Population Health, Centre for Ageing and Population Studies, UCL, London.

The term ‘frailty’ describes a condition characterised by loss of reserves causing vulnerability to a range of adverse outcomes after relatively minor illnesses.^1^ It has a reported prevalence of around 8.1% in people aged >50 years, with prevalence increasing to around 50% in people aged >90 years.^2^

Frailty can be a useful concept in primary care because it identifies older people at risk of a range of adverse outcomes, including loss of independence, serious falls, care home admission, and mortality. This prognostic information can be useful for making holistic, goal-oriented decisions in partnership with patients, families, and carers. However, alongside this prognostic information, there is also a growing evidence base for community-based interventions that can improve outcomes for older people living with frailty. These include ‘comprehensive geriatric assessments’ (CGA): a multidomain assessment of an older person’s medical, functional, social, psychological, and environmental needs to develop a shared care plan, which can sustain independence and reduce unplanned risk of admission to hospital.^3^,^4^ However, it needs to be targeted at appropriate cohorts of people to be feasible in the community. Alongside CGA, there is evidence that physical activity-based interventions, delivered alone or in combination with nutritional support, can improve outcomes in frailty.^5^

There is a lack of consensus on a standardised tool to measure and identify frailty, and in practice many methods can be used. This includes simple screening instruments, clinical assessments, the use of biomarker data, and electronic health records (EHRs).^6^ This editorial aims to improve the understanding of a new electronic frailty index, the eFI2, which is being incorporated into general practice EHR systems in England. It also explores ways in which the tool can be utilised in clinical practice among primary care clinicians.

## eFI2 compared to the original eFI

Several electronic frailty indices have been developed, some of which were independently validated, but most not applied in practice. One index, the electronic frailty index (eFI), identifies populations at risk of frailty and is calculated by the presence or absence of 36 individual factors, such as the presence of conditions such as falls, osteoporosis, and diabetes.^6^ It has been internally and externally validated. The original electronic frailty index was developed in 2016, derived from routinely collected primary care EHR data, and was supported by NHS England and incorporated into UK EHR systems used by primary care clinicians. Since then, it has been applied to local populations to predict who may be at risk of adverse outcomes in primary care as a result of frailty. Clinicians can use the eFI score to identify which people may need a further brief clinical assessment of frailty, using methods such as the clinical frailty scale, and may benefit from interventions to prevent adverse outcomes.

However, the original eFI had limitations including equal weighting of deficits, lack of time constraints (constraints were not applied to variables that could resolve over time), arbitrary cut-off points of frailty categories, and less emphasis on some deficits, such as mental health. A new version, the eFI2, was developed in 2025, to improve its performance and utility; and has also been internally and externally validated.^7^ The eFI2 addresses these shortcomings, for example, it allows components to be weighted for their contribution to the risk of frailty and can vary with time. Compared with the original eFI, it has improved performance for predicting frailty-related outcomes (new home care package, serious falls, nursing home admission, all-cause mortality), with very good calibration and discrimination statistics.

The major GP EHR software companies in England are replacing the original eFI with the eFI2 for national implementation and NHS England recommends using the eFI2 as part of its proactive care agenda.^8^ The tool will be widely available in 2025 to GPs and other clinicians working in primary care.

## How eFI2 can be used in clinical practice

There are clear gaps in understanding of the purpose of frailty tools such as the eFI in primary care^9^ and engagement is therefore essential to ensure it is used and found useful by primary care clinicians. There are also issues with the associated use of the term ‘frailty’ in patients’ medical records, which are prospectively viewable by patients. It has been shown that people do not like to self-identify with the term because of negative connotations related to ageing, stigma, and ‘weakness’,^10^ and this can potentially result in reduced motivation impacting self-management. Therefore, caution is needed before adding a diagnosis of mild, moderate, or severe frailty to patient records. If using the term, it should be explained to patients what it means in relation to their own personal health needs and goals.

In some cases, primary care clinicians felt the original eFI under- or overestimated frailty in older people.^9^ No tool is perfect and electronic tools such as the eFI and eFI2 are limited by what has been entered into electronic patient records, which may not be up to date. However, it is important to note that the eFI and eFI2 were not developed to be used as diagnostic tools but instead for population-level risk stratification to identify people at risk of adverse outcomes linked to frailty.

**Figure 1:**
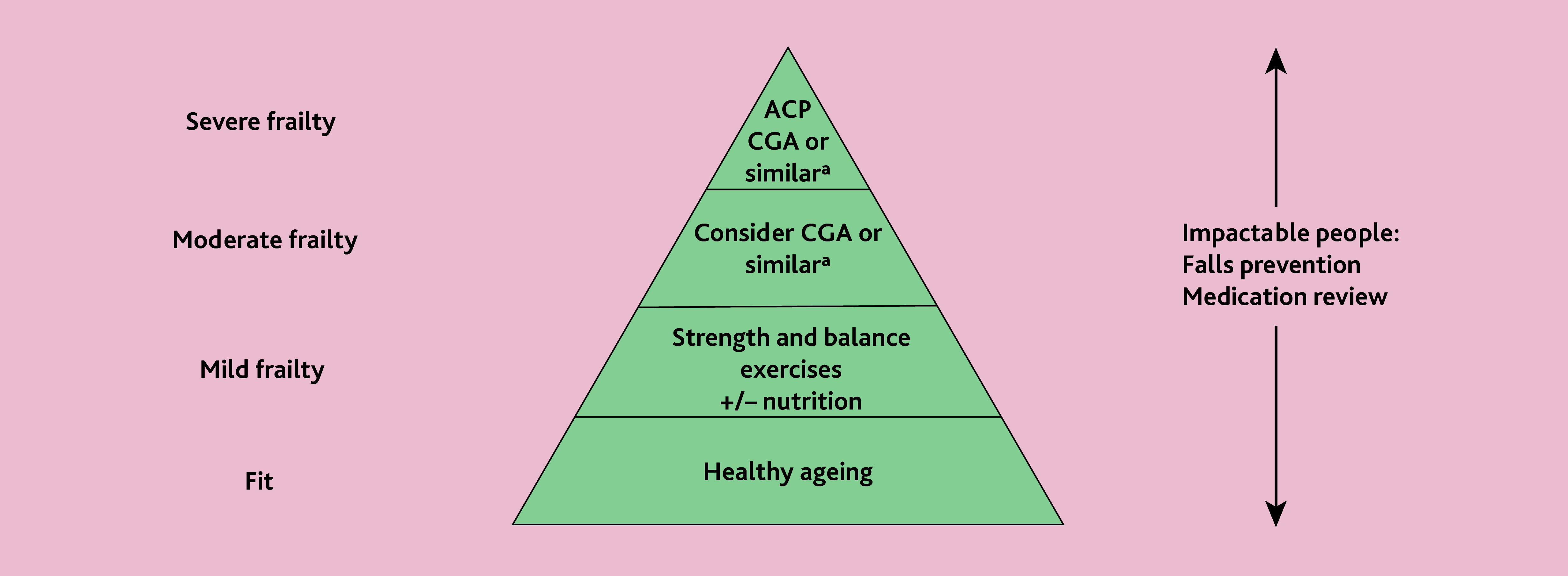
Interventions tailored according to frailty severity following a clinical assessment. ^a^CGA or similar holistic assessment and tailored management plan. ACP = advance care planning. CGA = comprehensive geriatric assessment.

In summary, clinicians should use the eFI2 to screen for people who may be mildly frail (0.09 ≤eFI2 score <0.16), moderately frail (0.16 ≤eFI2 score <0.24), or severely frail (eFI2 score ≥0.24). This should be followed by a clinical assessment to confirm the presence and severity of frailty, which would then result in tailored interventions according to severity in those for whom it may benefit.^7^ Figure 1 highlights a range of tailored interventions according to frailty severity that clinicians can consider and is based on the latest evidence and recommendations.^11^,^12^ It includes ‘impactable people’ across all groups, for example, someone who may be mildly frail but is at an increased risk of falling or has a high polypharmacy or anticholinergic medication burden. We acknowledge key components of a CGA or other similar holistic assessments with tailored management plans often take place in primary care frailty management; including a medical history, physical examination, functional assessment, cognitive assessment, mood screening, social support evaluation, and medication review.

The interventions chosen to be delivered will be guided by clinical judgement and depend on factors such as local population characteristics, the skill mix of the workforce to deliver care, infrastructure, and funding available. Ensuring staff ‘buy-in’ and utilising members of the multidisciplinary team is also important. For example: clinical pharmacists can conduct structured medication reviews; nurse practitioners can manage associated chronic conditions; physiotherapists can help prevent falls by teaching strength and balance exercises; occupational therapists can support independence by providing adaptive equipment; and social prescribers can support emotional wellbeing through health coaching and also signpost to local services. While further research is needed to determine if such approaches and models of care are cost-effective in primary care, the eFI2 has the potential to aid clinicians in identifying people with frailty to deliver more proactive care, through tailored interventions.

## Data Availability

The eFI2 model equation and associated code lists used to define variables are available for research use from Andrew Clegg. The eFI2 research team aim to make eFI2 available to suppliers of UK electronic health record systems, risk stratification software, and for use in NHS policy and commissioning under the terms of an agreed licence agreement. The eFI2 is licensed to suppliers of UK primary care electronic health record systems at no cost, on the basis that a premium charge is not subsequently applied to the end user.
